# Evolutionary history of host use, rather than plant phylogeny, determines gene expression in a generalist butterfly

**DOI:** 10.1186/s12862-016-0627-y

**Published:** 2016-03-08

**Authors:** Maria de la Paz Celorio-Mancera, Christopher W. Wheat, Mikael Huss, Francesco Vezzi, Ramprasad Neethiraj, Johan Reimegård, Sören Nylin, Niklas Janz

**Affiliations:** Department of Zoology Ecology, Stockholm University, Svante Arrheniusväg 18 B, 106 91 Stockholm, Sweden; Science for Life Laboratory, Stockholm, Sweden

**Keywords:** Coevolution, Speciation, Gene expression, Host shift, *Vanessa cardui*, Pest species

## Abstract

**Background:**

Although most insect species are specialized on one or few groups of plants, there are phytophagous insects that seem to use virtually any kind of plant as food. Understanding the nature of this ability to feed on a wide repertoire of plants is crucial for the control of pest species and for the elucidation of the macroevolutionary mechanisms of speciation and diversification of insect herbivores. Here we studied *Vanessa cardui*, the species with the widest diet breadth among butterflies and a potential insect pest, by comparing tissue-specific transcriptomes from caterpillars that were reared on different host plants. We tested whether the similarities of gene-expression response reflect the evolutionary history of adaptation to these plants in the *Vanessa* and related genera, against the null hypothesis of transcriptional profiles reflecting plant phylogenetic relatedness.

**Result:**

Using both unsupervised and supervised methods of data analysis, we found that the tissue-specific patterns of caterpillar gene expression are better explained by the evolutionary history of adaptation of the insects to the plants than by plant phylogeny.

**Conclusion:**

Our findings suggest that *V. cardui* may use two sets of expressed genes to achieve polyphagy, one associated with the ancestral capability to consume Rosids and Asterids, and another allowing the caterpillar to incorporate a wide range of novel host-plants.

**Electronic supplementary material:**

The online version of this article (doi:10.1186/s12862-016-0627-y) contains supplementary material, which is available to authorized users.

## Background

Insects and the flowering plants they feed upon comprise the majority of described species and total biodiversity on Earth, yet we know little about why these clades dominate our planet. Although the vast majority of these insect species are specialist feeders, consuming only a few closely related plant species, modern phylogenetic analyses reveal that most specialist clades are millions of years younger than their plant hosts [1–3]. Therefore, insect specialists must typically derive from ancestors that shifted between host plants over evolutionary time, rather than solely originating via coevolution with their host-plants. While such shifts in host use have been significantly associated with increases in diversification rate, and are argued to have played a significant role in the generation of herbivorous insect diversity [4], very little is known about their origins in natural systems. Thus, one way to understand the paradox of most herbivorous insects being specialized but on a diverse set of host plants is to investigate the origins of major shifts in host use.

Here, we test a fundamental hypothesis about the origin of a major change in host-plant utilization. Traditionally, molecular genetic studies of host shifts have focused upon key adaptations in species that have specialized on hosts over millions of generations since they initially started using them (e.g. papilionid butterflies using P450s or pierid butterflies using nitrile specifier protein (NSP) for detoxification of hosts) [5, 6]. In contrast, here we focus upon an herbivorous insect that recently evolved to feed upon an extended range of novel host plants, while retaining the ability to feed upon a core set of ancestral host plants. We view this as a potential first step in a major host shift, and this rare feature allows us to test whether the digestive physiology of feeding (as reflected in gene expression) is shaped more by the evolutionary history of the plants they are feeding upon, or the evolutionary history of the insect-plant associations. A clear association with plant phylogeny would suggest that plant chemistry shapes larval digestive evolution, as related plants would elicit a similar digestive response. This is the predominant view of plant-insect interactions ever since coevolution was first envisioned [7] and is supported by the fact that host use typically follows strong and conservative taxonomical patterns, with hosts being largely restricted to specific plant taxa over very long time spans [5, 6, 8–11]. Indeed, niche conservatism with strong phylogenetic signal is a universal aspect of ecological species associations [12], meaning that it is a reasonable null hypothesis even though exceptions certainly frequently occur – e.g. because of convergent chemical similarities [7]. In contrast, a pattern reflecting the evolutionary history of the associations, where gene expression more strongly recognizes the differences between ancestral vs. novel plants than their phylogenetic relationships, would suggest another categorization of insect adaptations in digestive physiology. Specifically, we suggest that generalist species will be found to possess “general-purpose” physiological adaptations enabling them to cope with novel plants irrespective of their precise chemical characteristics.

The butterfly *Vanessa cardui* (Nymphalidae; Lepidoptera) is on the threshold of becoming a true generalist and potential agricultural pest [13–17]; it is a cosmopolitan migrant species that makes annual, multi-generational, mass migrations up to 15,000 km long from Africa to Northern Europe, and whose larvae feed on more than 10 Orders of angiosperm plants [18, 19]. As most butterflies feed on host plants in a single plant family, *V. cardui* is arguably the most polyphagous of all butterflies [11] and therefore presents a unique opportunity to gain insight into the process that enables the relatively unconstrained host use commonly seen in insect pests. For example, most of the close relatives of *V. cardui* (Nymphalini, 20 species) feed on hosts from a couple of plant families or feed on various combinations of a limited set of plant groups [20] such as the ”urticalean rosids” (e.g. Urticaceae), which are ancestral hosts for the subfamily of Nymphalinae which is approximately 45 million years old [21]. Thus, *V. cardui* has a host range that breaks the norm of host-plant use in the Nymphalini; although some plants in its repertoire are shared with extant relatives, *V. cardui* has also uniquely colonized a wide array of plants not used by other Nymphalini (and rarely among other butterflies) [11]. To explain this pattern, we hypothesize that *V. cardui* caterpillars are using two different kinds of physiological adaptations which will be reflected by different correlated sets of gene expression. The first type allows this insect to feed on plants that they share with relatives and these commonly used ancestral host plants will be referred to hereafter as the “core repertoire” of host plants. The other type allows *V. cardui* using novel plants that no other Nymphalini butterflies feed upon and these plants will be referred to as the “extended repertoire”.

In order to test this hypothesis, we obtained transcriptome profiles of several different tissues from *V. cardui* caterpillars reared on three species each from the core and extended groups of plants. Gene expression profiles across four tissues were then tested to see whether variation was explained better by the core and extended host-plant categories, or the phylogenetic relatedness among host-plants. We then compared these results with measures of larval performance and adult preference (oviposition) on these plants to assess the concordance across life history stages.

## Results

### Transcriptome hypothesis testing

To test whether the evolutionary history of host-plant use rather than plant phylogeny dictates patterns of gene expression in caterpillars, we assessed a model determining the source of variability in the caterpillar transcriptional dataset from labial gland, fat body, gut and Malpighian tubules. After quality filtering, we obtained gene expression profiles of each tissue from caterpillars fed on six different host-plants (biological triplicates with > 9 M reads each). As exemplars of the core and extended groups, we used *Malva silvestris*, *Cirsium arvense* and *Urtica dioica*, for the former, and for the latter *Cynoglossum officinale*, *Plantago lanceolata*, and *Ranunculus acris*.

**Fig. 1:**
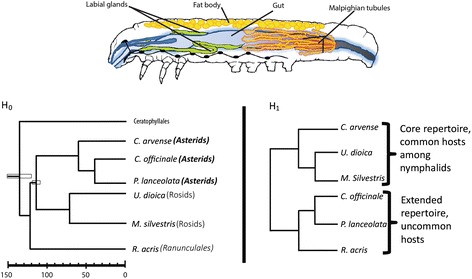
Caterpillar internal anatomy and hypothetical dendograms grouping *V. cardui* transcriptional response on the different plants. Estimates in Ma for the age of Ranunculales, Rosids and Asterids based on those published elsewhere [35–37]. Caterpillar drawing was taken and modified from a previous publication [38]. Permission granted for the use of the drawing by John Wiley & Sons, Inc. (All rights reserved. No part of the drawing may be reproduced, stored in a retrieval system, or transmitted, in any form or by any means, electronic, mechanical, photocopying, recording or otherwise, except as permitted by the UK Copyright, Designs and Patents Act 1988, without the prior permission of the publisher.)

In our model, which also included *tissue* as variable, *plant use* reflected the grouping of core vs. extended. Our null hypothesis was that the variability in tissue-specific expression would reflect plant relatedness. The plant relatedness was named *plant phylogeny* in our model. According to the null hypothesis *U. dioica* and *M. silvestris* are more closely related to each other (Rosids), *P. lanceolata*, *C. officinale* and *C. arvense* represented another phylogenetic group (Asterids) and *R. acris* is the outgroup belonging to the distant Ranunculales (Fig. 1).

**Fig. 2:**
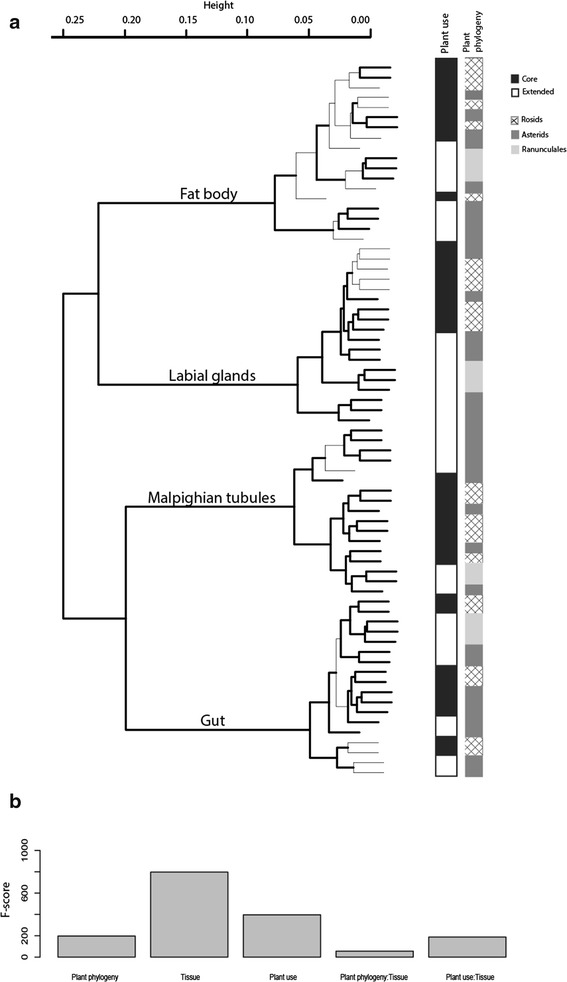
Statistical data analyses of the tissue-specific transcriptomes from caterpillars reared on six host-plant species. *Hierarchical clustering with bootstrapping* **a**. Each branch in the dendogram represents the expression profile of a transcriptome. Each transcriptome is a biological replicate represented in turn by the read-count data generated from 9 million reads summed by ortholog (12 591 orthologs). Transcriptomes in a given cluster have similar expression profiles. Thick-black branches indicate p-value and bootstrap probabilities > 90 for a given cluster. The vertical bars next to the dendogram indicate the categories under *plant use* or *plant phylogeny* for each transcriptome. *Plant use*: core repertoire in black and extended repertoire in white. *Plant phylogeny*: Rosids in grey linear gradient, Asterids in solid dark grey and Ranunculales in solid light grey*.* Each transcriptome perfectly clusters by tissue and within tissue clusters more strongly according to plant use than plant phylogeny. *ANOVA testing the effect of tissue, plant use and plant phylogeny and interactions of the factors* **b**. All factors and their interactions were significant (P < 2.2 e^−12^). F-scores obtained from ANOVA analysis for factors are graphed. The variable *tissue* was the most significant explaining the variability in the gene-expression data, followed by *plant use* and last *plant phylogeny*

Using the partial least squares discriminant analysis (PLS-DA) model, after grouping of the gene-expression data from caterpillars by *tissue*, *plant use* explained most variation in our dataset (Table 1). In contrast, the data is poorly predicted by *plant phylogeny*, as this variable either results in a non-predictive model or non-significant predictive components in the model (Table 1). Another type of supervised multivariate analysis produced similar results and is provided in an additional table (see Additional file 1). Hierarchical clustering revealed a clear separation of the samples by caterpillar *tissue* with the Malpighian tubules and gut transcriptomes more similar to each other in contrast to the group including the labial gland and the fat-body transcriptomes (Fig. 2A). However, the gene expression profiles within each tissue group followed *plant use* and to a lesser degree the *phylogeny* of the plant (Fig. 2A). Not surprisingly, *plant phylogeny*, reflecting similarity of chemical composition of related plants, accounts in some degree for the variability of the transcriptional response in caterpillars. We could verify these observations when an ANOVA was conducted on the data (Fig. 2B). The variable *tissue* was the most significant explaining the variability in the gene-expression data, followed by *plant use* and last *plant phylogeny*. Some of the variability in the data cannot be solely explained by one or the other variables alone. Actually, the interaction between *tissue* and *plant use* explain a good proportion of the variability in the data. Taken together, these results suggest that digestive physiological adaptations in caterpillars of the species *V. cardui* discriminate primarily their host-plants based upon their history of evolutionary interaction with them, rather than the evolutionary relationships among the plants themselves.

**Table 1:** Multivariate statistical analyses of the explanatory variables for the caterpillar-expression dataset

Model description	PC	Samples	R^2^	Q^2^	*P* value*
Tissue	4	71	0.985	0.982	0
Plant use	6	71	0.93	0.804	2.7e-13
Plant phylogeny	2	71	0.341	0.196	5.4e-5
Plant use within gut	4	18	0.991	0.875	0.01
Plant use within Malpighian tubules	3	17	0.977	0.83	0.01
Plant use within fat body	4	18	0.992	0.929	0.005

The analyses tested the goodness of fit (R^2^) and predictive ability (Q^2^) of *tissue*, *plant use* and *plant phylogeny* as explanatory variables. The significance of the PLS model was tested using a cross-validated ANOVA and the p-value metric is presented in the table (*). The number of samples is also provided for each corresponding model and the number of predictive components (PC)

The heatmaps provided in an additional figure illustrate the tissue-specific profiles of those differentially expressed genes in response to the core and extended repertoire of plants (see Additional file 2). The transcriptomic profiles reveal a higher degree of variation in the response to *U. dioica* in the gut of caterpillars and variability in the gene-expression response towards both *U. dioica* and *P. lanceolata* in the fat body and Malpighian tubules.

### Functional annotation analyses

Analyses using the functional annotation of tissue level expression also showed an effect of evolutionary history of *plant use* rather than *plant phylogeny*. Gene set enrichment analyses were only significant when comparing the transcriptional responses between core vs. the extended repertoire of plants in two tissues, the fat body and gut of caterpillars (Table 2). Gene expression in the fat body was enriched for genes involved in oxidoreductase activity and metabolism of carboxylic acids and in the gut it was enriched for genes encoding proteins integral to the membrane. A complete list of those genes involved in oxidoreductase activity in the fat body is provided in an additional table (see Additional file 3).

**Table 2:** Gene-ontology (GO) categories overrepresented among the differentially expressed genes for the variable *plant use*. Gene expression data were obtained from different tissues from caterpillars reared on six different plants grouped according to either their evolutionary history of association with nymphalids (*plant use*)

Variable	Tissue	GO Categories	GO ID	Term	P-value (adjusted)
Plant use	Fat body	Biological Process	GO:0044281	small molecule metabolic process	0.0040
			GO:1901564	organonitrogen compound metabolic process	0.0040
			GO:0055114	oxidation-reduction process	0.0049
			GO:0044710	single-organism metabolic process	0.0106
			GO:0019752	carboxylic acid metabolic process	0.0112
			GO:0006082	organic acid metabolic process	0.0142
			GO:0043436	oxoacid metabolic process	0.0142
		Molecular Function	GO:0016491	oxidoreductase activity	0.018
	Gut	Cellular Component	GO:0016021	integral to membrane	0.047
			GO:0031224	intrinsic to membrane	0.047

Genes that were differentially expressed between core vs. extended in the different tissues are mentioned in an additional list, with the highest fold change in putative regulators of cell cycle and DNA replication (see Additional file 4). Other differentially expressed genes were involved in digestive, detoxifying and metabolism-related functions. Oxidoreductases, substrate transporters and a gustatory receptor were also up-regulated in caterpillar tissues in response to the extended repertoire of plants.

### Butterfly oviposition preference and caterpillar performance

**Fig. 3:**
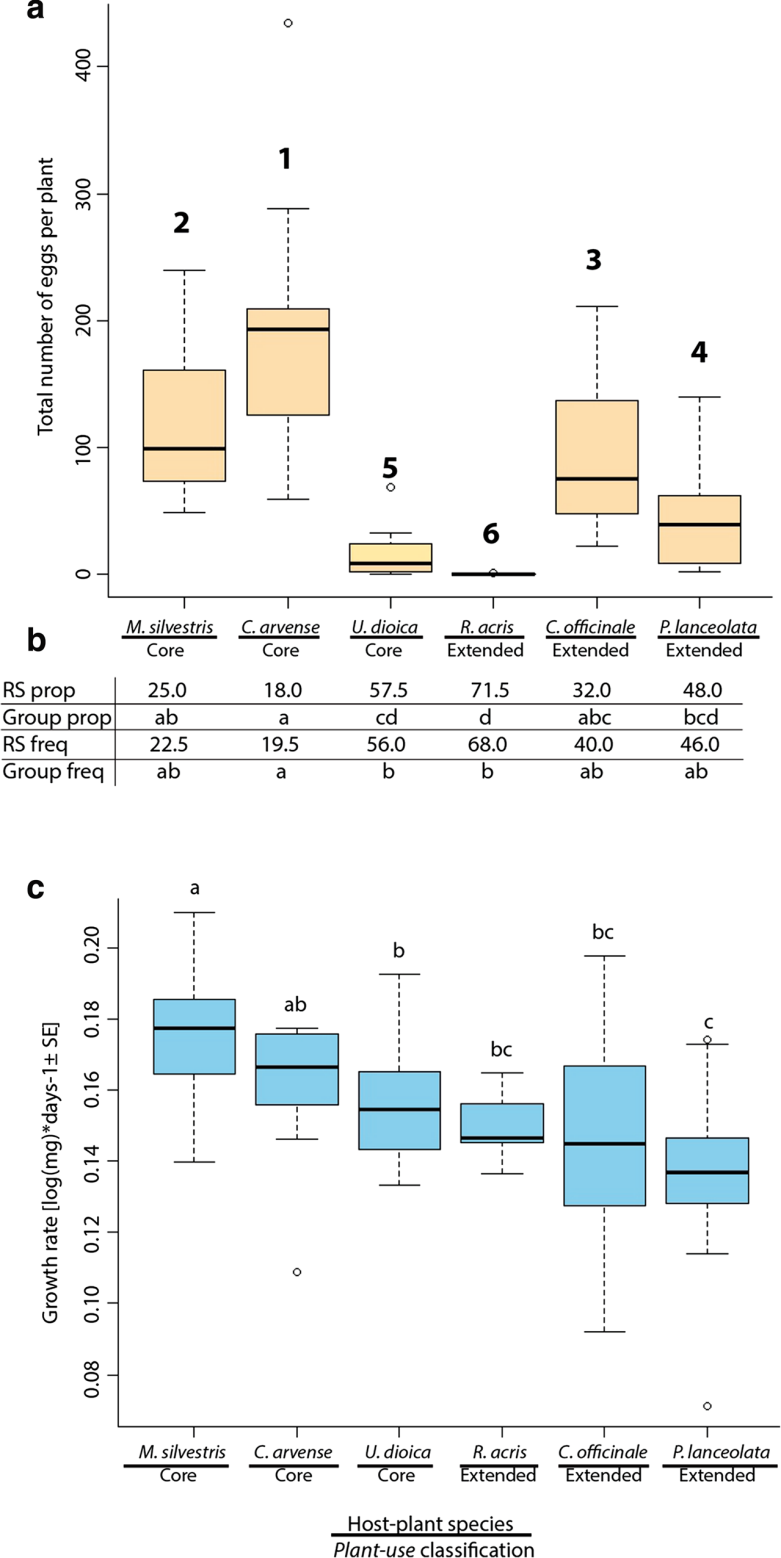
Oviposition preference by female adults and larval performance on the six host-plant species in the study. *Box-and-whisker plots of the total number of eggs laid per female on each host-plant species* **a**. Host-plant species in the study are on the x-axis along with their classification based on *plant use* (core or extended). *Ranking of plants according to female preference for oviposition* **b**. Two kinds of rank-ordering of plants per female butterfly were performed; one based on proportions and the other on cumulative frequency of preferred plant for each paired choice (preference scores). Results presented are based on multiple comparison tests (k = 6, n = 12) after significant Friedman-rank sum (RS) based on proportions (RS prop) or frequencies (RS freq). Groups (Group prop and Group freq) are designated by letters so that plants with rank sums without the same letter are significantly different from each other determined by post-hoc correction (P < 0.04). Ranking of plants ranged from 1 (lowest RS) to 6 (highest RS) is shown above each plot in A. *Performance of V. cardui caterpillars on six plants selected based on their history of interaction with nymphalids through evolutionary time* **c**. Growth rate to pupation [log(mg)*days-1 ± SE] per plant. Rates without the same letter are different from each other according to multiple after a significant effect of plant on growth rate (*Χ*2 = 59.06, df = 5, P = 1.901e-11)

Assays of female-oviposition preference indicated that although there was some variability in the total number of eggs laid by each female per plant, females laid the highest number of eggs in *C. arvense* and the lowest in *R. acris* (Fig. 3A). The experimental design did not allow us to compare the total number of eggs per plant per female directly. Rather, two ranking methods were used for the analysis of choice experiments. Based on these methods, adult females discriminated among plants and this result was consistent regardless of ranking method used (proportion-based ranking: Q = 49.83, df = 5, p < 0.001 vs. frequency-based ranking: Q = 42.34, df = 5, p < 0.001). The multiple comparison procedure showed that the six plants assayed can be grouped in three main categories based on preference: high, intermediate, and low (Fig. 3B). While two of the core repertoire plants (*C. arvense*, *M. silvestris*) were highly preferred choices for oviposition, the third core species (*U. dioica*) was ranked among the least preferred group of plants along with a member of the extended repertoire (*R. acris*). In the intermediate group were *C. officinale* and *P. lanceolata*, although these two plants are part of the extended repertoire of *V. cardui*. Thus, female oviposition preference was not concordant with plant phylogeny and only partially with the core vs. extended categorization (*plant use*).

The growth rate of caterpillars (performance) was different depending on the host plant (*Χ*^2^ = 59.06, df = 5, P = 1.901e-11)(Fig. 3C). As observed in the oviposition preference, we observed three main groups based on performance differences. Caterpillar performance appeared to be more concordant with *plant use*. That is, caterpillars performed best when using plants in the core group and worse when using plants in the extended group. However, two plants belonging to the core group and two plants from the extended group are no different in terms of their effects on caterpillar performance and can be regarded as “intermediate” in terms of their suitability as food. Two hosts are highly ranked for both oviposition preference and larval performance and they do not belong to the same plant phylogenetic group i. e. *M. silvestris* and *C. arvense* (Fig. 3).

*P. lanceolata*, an asterid from the extended repertoire of *V. cardui*, was a better food source for the caterpillars than what could be expected from the oviposition choice of the adults. In contrast, *U. dioica* does not rank among the most preferred plants for oviposition but ranks as intermediate in host quality for caterpillar performance. Another inconsistency between the adult preference and the caterpillar performance on the studied plants is that *C. officinale* was ranked the third most preferred plant for female oviposition but the plant is just ranked above the worst host for the larvae in terms of growth rate (Fig. 3). Conversely, *R. acris* was not the worst host plant but the least preferred plant for oviposition by the adult female.

## Discussion

Transcriptome variation among larvae feeding on six different host-plants was primarily determined by the evolutionary history of their ancestor’s interactions with these plants, rather than the evolutionary relationship among the plants themselves. Regardless of our analysis approach, after accounting for the strong expression variation among the four tissues studied, grouping of expression variation by *plant use* consistently explained 50 % more variation than *plant phylogeny* and obtained better support as an explanatory variable in terms of predictive ability of the model. This result is completely unexpected from traditional insect-plant theory, where the phylogenetic relationship among plants is the de facto proxy used for chemical and morphological similarity among host-plants ([7] and references herein). Rather, our results support our prediction that such a generalist butterfly also has general-purpose adaptations for coping with novel hosts in a way which is less constrained by chemistry.

The only way to reconcile our results with prevailing theories would be if the core and extended host categories are in fact characterized by convergent chemical similarities within each group that are stronger than those similarities that correlate with the phylogenetic patterns. Given the evolutionary distance between the studied host-plant species, we consider this highly unlikely as a general explanation. The monophyletic clades of Asterids and Rosids last shared an ancestor nearly 100 Ma. Each contains multiple orders of plants, numerous families, and they are the two most species-rich groups of Eudicots. Given that nearly all herbivorous insect species have a host range that does not extend beyond the level of a plant family, strong chemical similarities between these clades are very unlikely. For the category of extended hosts, this problem is further magnified as Asterids and Ranunculales are even more evolutionarily divergent. It does remain a possibility that the similarity in gene expression among the core hosts are at least partially explained by convergent chemical similarities, which would account for their retention as hosts over a long period of evolutionary time. However, among the extended hosts we see no reason at all to expect such similarities.

In contrast to the transcriptional data, we could not find a clear separation between the core and extended host categories of plants when we inspected adult preference for oviposition, i.e. core hosts were not preferred over extended hosts. This mismatch between caterpillar gene-expression and adult preference reflects that the adult stage is less conservative in its host use and more opportunistic while the caterpillar is restrained by chemical adaptation to the host. We also found a small effect of history of plant use in terms of larval performance, as feeding on novel hosts in the extended host repertoire did not necessarily lead to lower growth rate.

Our results have major implications at the molecular and macroevolutionary levels. At the molecular level, we suggest that the distinct expression patterns of *V. cardui* larvae feeding on core vs. extended groups of plants may be the consequence of two distinct modes of transcriptional regulation. We posit that these different sets of gene expression allow them to retain their ancestral capability to consume plants in the orders of Rosids and Asterids, while also incorporating novel hosts to which no long-term adaptation has taken place. This means that even major host shifts are potentially possible, i.e. if a future descendant of *V. cardui* were to specialize on one of these novel hosts. A gene expression set allowing use of distantly related plants may in fact be one of the reasons that ancestors of this group of butterflies were originally able to feed on plants in both the orders of Rosids and Asterids. In line with our findings, it has been found both in Australian soapberry bugs [22, 23] and Californian butterflies [24] that generalists are more likely to colonize introduced exotic plants. Nearly all butterflies specialize on closely related host-plants, meaning that the evolution of extremely opportunistic species is rare in butterflies (Papilionoidea) as compared to other Lepidopteran clades, which have globally significant agricultural pests. We feel that our findings here, rather than being specific to *V. cardui*, suggest that the two distinct modes of coping with host plants may be a significant and un-studied factor in the evolutionary origin of pest species. Such correlated sets of gene expression are consistent with West-Eberhard’s (2003) broad concept of modularity [25] as a central factor when genotypes and phenotypes evolve. Even though the gene expression patterns at this point overlap to a large extent, selection may act over time to produce more distinct modules of co-adapted traits, or alternatively one of the two modes may be lost.

At the macroevolutionary level, we have proposed elsewhere that a generalist stage represents a plastic but evolutionarily transient state that could give rise to increased rates of diversification [4, 26]. The current host-plant breadth of *V. cardui* is on the scale of being a true opportunistic generalist, while also being a cosmopolitan migrant. Given the long observed positive correlation between geographic distribution and speciation rate [27], the *V. cardui* lineage appears evolutionarily poised to increase its diversification rate. Should the resulting daughter species follow the predominant herbivorous insect trend and specialize on a limited set of hosts, such events would eventually result in butterfly species feeding on a few closely related host-plants and being millions of years younger than the host-plant lineages. We consider these events to be highly likely, as they are driven by the most common observations in the world around us. Our contribution here is to document that this process may be driven by a modularity of mechanisms, and to suggest that such modularity likely facilitates the transition between specialists vs. generalist states over evolutionary time.

In butterflies the trend towards specialization seems too strong to permit the evolution of the kind of extremely opportunistic species seen in some moth taxa, species that also are prone to become important pests in agriculture and forestry. However, the apparently fundamental distinction revealed here between transcriptional regulatory modes for dealing with core hosts and opportunistically used extended hosts may well give important clues towards an improved understanding of the evolutionary origin of pest species.

## Conclusion

Recent phylogenetic analyses suggest that the diversity of insect herbivores has evolved not only through an arms-race with their host-plants over long time periods, but from ancestors that were able to shift onto novel host-plants. Understanding an insect’s ability to shift onto novel plants is therefore central to understanding the origins of insect biodiversity and the nature of herbivores with a broad host range. We hypothesized that two sets of expressed genes, associated with different modes of digestive physiology, are needed to initially achieve generalist herbivory: one adapted for feeding on a group of plants forming a common ancestral repertoire for an insect taxon and one adapted to cope with new hosts regardless of plant chemistry. We found evidence to support this hypothesis when analyzing the tissue-specific transcriptomes from caterpillars of a potential insect pest feeding on six different host-plants.

## Methods

### Plant and Insect Material

The experiments were conducted during autumn 2011 (gene expression) and summer 2012 (oviposition preference and larval performance). The hosts (*U. dioica*, *M. silvestris*, *C. arvense*, *C. officinale*, *R. acris* and *P. lanceolata*) were planted from seed and cultivated (average 25 °C and a 12 h day length) for 3 months before they were used as food for the larvae. The use of these plants complies with institutional, national and international guidelines including the Convention on the Trade in Endargered Species of Wild Fauna and Flora. Butterfly larvae and pupae of the species *V. cardui* were obtained from a commercial supplier during autumn 2011 (World Wide Butterflies [www.wwb.co.uk]). No specific field work or ethical permission to collect and use the caterpillars and butterflies in this study was necessary. This population was maintained under laboratory conditions (25 °C; LD 18:6) avoiding full-sib mating over the winter and crossed with two new batches of butterflies also obtained commercially (World Wide Butterflies [www.wwb.co.uk] and Heart of England Butterflies [www.heartofenglandbutterflies.com]) in the spring of 2012. The adults of the first generation of this intermixed population were used to assess the female preference of plant for oviposition. The second generation was used to evaluate larval performance on the different plants. Progeny from six females (gene expression) and from four females (larval performance) were reared on the plants mentioned above (mean temp. 26 °C; mean RH 38.4 %; LD 18:6) following a split-brood design. That is, ten neonates (gene expression) and thirty neonates (larval performance) from each family were transferred immediately after hatching to individual plastic cups containing leaves of each different plant. Leaves were kept moist using a wet cotton ball at their base and replaced every day. Larvae fed on their corresponding hosts until the second day after molting into their fifth instar for the gene expression experiment. To assess larval performance, the caterpillars were reared until they reached the adult stage. An additional file describes the methodology followed for the oviposition and larval performance experiments (see Additional file 5).

### Read-count data

The methods conducted for caterpillar RNA isolation, quality assessment and sequencing are described in an additional file (see Additional file 6). The RNA sequences obtained were assembled de novo using Trinity software [28]. Redundant sequences in the transcriptome assembly (TA) generated by Trinity were clustered using the program CD-HIT 4.5.4 (Cluster Database at High Identity with Tolerance) [29]; thereafter this clustered assembly was used. Using Blast (basic local alignment search tool) (National Center for Biotechnology Information, Bethesda MD) the TA sequences were compared at the amino acid level to a non-redundant predicted gene set (PGS) of the nearest genome, *Heliconius melpomene*. The non-redundant PGS of *H. melpomene* was generated using CD-HIT 4.5.4 clustering using default settings on the full *H. melpomene* PGS. In order to measure the quality of the Trinity-generated TA, these Blast-inferred orthologs were used to determine the fraction of PGS assembled and the length of the assembled region. The length of the TA-sequence that is aligned, divided by the full length of the best hit ortholog, namely Ortholog Hit Ratio (OHR), has been previously used as a measure of TA quality [30]. Two OHR metrics were generated. First, the longest TA-sequence for each of the 12,591 *H. melpomene* genes was determined (longest OHR). Second, the full length covered of each of these genes was determined by using multiple sequences (sum OHR). Gene expression was quantified using the summed per ortholog number of RNA-Seq reads with the aid of an in-house script developed in Python (Python Software Foundation, DE) using the mapping software NextGenMap 0.4.10 [31] on default parameter settings. Gene ontology was obtained through lifting over the annotation of the non-redundant PGS for *H. melpomene* to the Blast-inferred orthologs. The methodology for gene annotation and enrichment was also included in an additional file (see Additional file 6).

### Description of gene-expression data

We utilized hierarchical clustering with bootstrapping (10 000 bootstraps) as a method of unsupervised learning to describe patterns of gene expression [32]. The analysis was implemented using the statistical program R 3.0.3 [33] on read-count data (summed per ortholog), log-transformed and scaled using the Trimmed Mean of M values procedure [34]. The R code used to analyze the data is available at <https://github.com/hussius/butterfly>.

### Hypothesis testing: Evolutionary history of plant use vs. plant phylogeny

Since the variables plant use (by nymphalids) and plant phylogeny are different properties of a given plant they are highly correlated. PLS-DA is an appropriate option for analysis since it is a method for creating multiple regression equations even if the variables are correlated. Therefore, in order to test whether profiles of gene expression will group into the core and extended diet categories, rather than into groups reflecting plant phylogenetic relatedness (Fig. 1), we applied the PLS-DA and an extension of this analysis (orthogonal partial least squares regression) utilizing the SIMCA 13.0.3 software (www.umetrics.com, Umeå, Sweden). Additionally, we used R 3.0.3 to conduct another method of data analysis (ANOVA) to verify variable correlation and test linear models to address whether the variables tissue, plant use and plant phylogeny are significant factors explaining the variability in the normalized data.

### Availability of supporting data

The data set supporting the results of this article is available in the ArrayExpress repository, [http://www.ebi.ac.uk/arrayexpress/experiments/E-MTAB-3861].

## Additional files

Additional file 1: Is a table showing the statistics of the OPLS analysis on the variables tissue, plant use and plant phylogeny explaining the pattern of expression of the caterpillar genes (PDF 255 kb) Additional file 2: Shows heatmaps for all differentially expressed genes per caterpillar tissue towards the core and extended repertoire of plant species used in the study (PDF 501 kb) Additional file 3: Is a table listing the transcripts under the “oxidoreductase” GO category in the fat body of caterpillars in response to the extended repertoire of plants (PDF 99 kb) Additional file 4: Is a table listing tissue-specific transcripts up- regulated in response to the extended repertoire of plants (PDF 370 kb) Additional file 5: Provides the details of the methodology followed when conducting the oviposition preference and larval performance experiments (PDF 259 kb) Additional file 6: Provides the methodology for sequencing of RNA and gene annotation and enrichment of the transcriptional dataset. (PDF 294 kb)

## Abbreviations

NSP: Nitrile-specifier protein
PLS-DA: Partial least squares discriminant analysis
OPLS: Orthogonal partial least squares
ANOVA: Analysis of variance
Ma: Million years
RH: Relative humidity
CD-HIT: Cluster database at high identity with tolerance
OHR: Ortholog hit ratio
GO: Gene ontology
