# Supplementary material for: Evolutionary history of host use, rather than plant phylogeny, determines gene expression in a generalist butterfly
Source: BMC Evol Biol. 2016 Mar 8;16:59. doi: 10.1186/s12862-016-0627-y (PMC4782335; doi:10.1186/s12862-016-0627-y)
Supplement: Additional file 1: — Is a table showing the statistics of the OPLS analysis on the variables tissue, plant use and plant phylogeny explaining the pattern of expression of the caterpillar genes (PDF 255 kb) [file 12862_2016_627_MOESM1_ESM.pdf]

Additional file 1. Results of an O-PLS analysis on the variables tissue, plant use and plant phylogeny explaining caterpillar gene expression.

**Model description**	**# PC**	**Sample #**	**R^2**	**Q^2**	***P* value***
Tissue	3+**2**	71	0.993	0.989	0
Plant phylogeny	2+**10**	71	0.983	0.701	6.3e-14
Host plant use	1+**6**	71	0.976	0.787	5.1e-14
Host plant use within gut	1+**6**	18	1	0.886	0.017
Host plant use within fat body	1+**6**	18	1	0.927	0.004
Host plant use within malpighian tubules	1+**2**	17	0.977	0.757	0.011
Host plant use within labial gland	1+**4**	18	0.999	0.802	0.018
Plant phylogeny within gut	2+**6**	18	0.999	0.775	0.05
Plant phylogeny within fat body	2+**4**	18	0.969	0.625	0.08
Plant phylogeny within malpighian tubules	2+**2**	17	0.94	0.66	0.034
Plant phylogeny within labial gland	2+**1**	18	0.8	0.452	0.024

Multivariate statistical analyses testing the goodness of fit (R2) and predictive ability (Q2) of plant use and plant phylogeny as explanatory variables for the caterpillar-expression dataset. The significance of the orthogonal partial least squares (O-PLS) model was tested using a cross-validated ANOVA and the p-value metric is presented in the table (*). The number of samples are also provided for each corresponding model and the number of predictive components (PC) including those that do not contribute to class separation (in bold).
