# Supplementary material for: Evolutionary history of host use, rather than plant phylogeny, determines gene expression in a generalist butterfly
Source: BMC Evol Biol. 2016 Mar 8;16:59. doi: 10.1186/s12862-016-0627-y (PMC4782335; doi:10.1186/s12862-016-0627-y)

Additional file 2. Heatmaps of all differentially expressed genes per caterpillar tissue in response to the core and extended repertoire of plant species used as food. The color scale represents the expression level in normalized log-counts 0 to 15. Each end of the branch per dendogram represents a replicate and squares underneath it indicate the plant species (color-coded) and the plant classification according to the variable *plant use* where the core repertoire is represented by black squares and the extended by white.

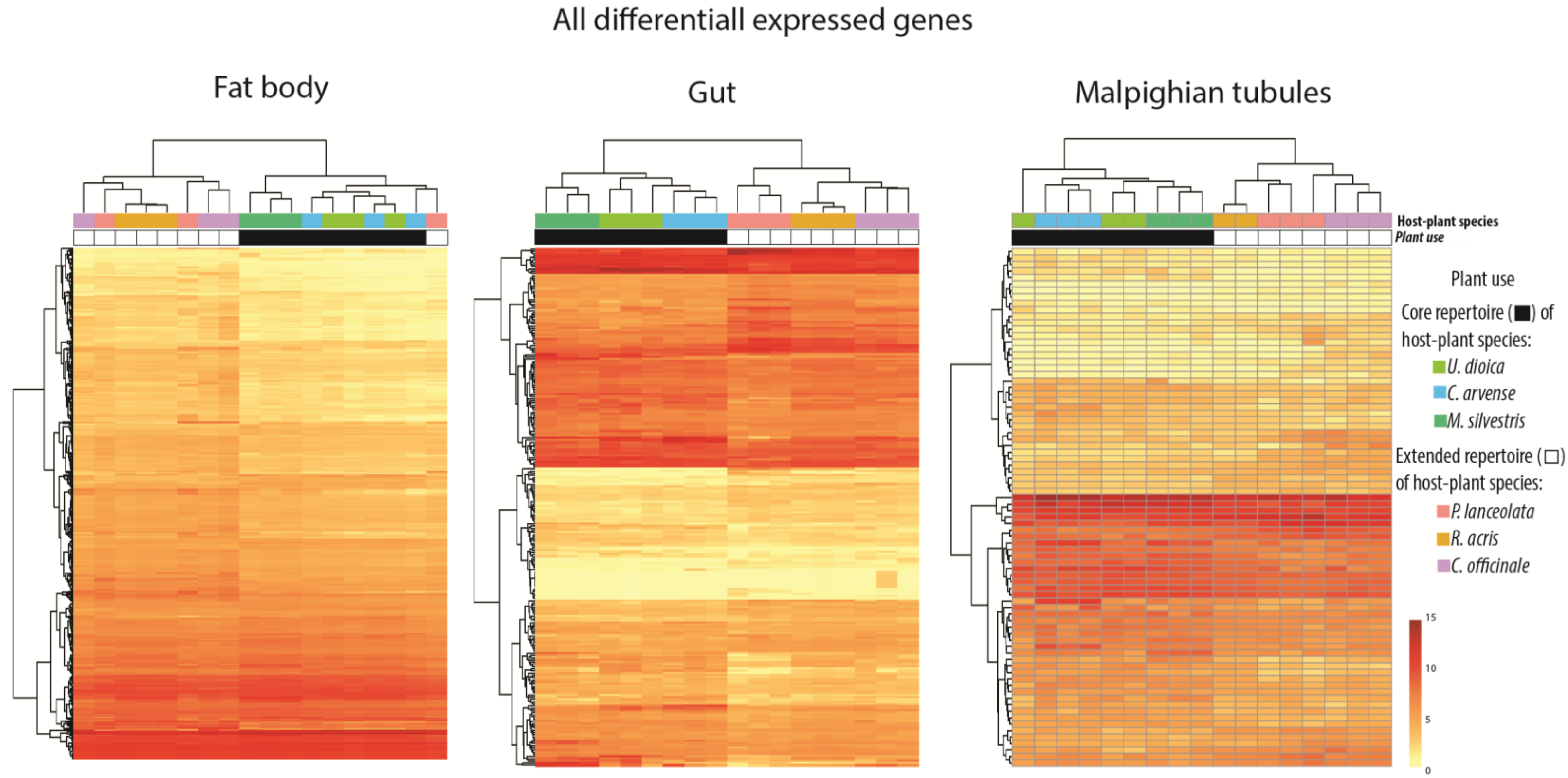

Supplement: Additional file 2: — Shows heatmaps for all differentially expressed genes per caterpillar tissue towards the core and extended repertoire of plant species used in the study (PDF 501 kb) [file 12862_2016_627_MOESM2_ESM.pdf]
