# Supplementary material for: Evolutionary history of host use, rather than plant phylogeny, determines gene expression in a generalist butterfly
Source: BMC Evol Biol. 2016 Mar 8;16:59. doi: 10.1186/s12862-016-0627-y (PMC4782335; doi:10.1186/s12862-016-0627-y)
Supplement: Additional file 3: — Is a table listing the transcripts under the “oxidoreductase” GO category in the fat body of caterpillars in response to the extended repertoire of plants (PDF 99 kb) [file 12862_2016_627_MOESM3_ESM.pdf]

Additional file 3. List and regulation of transcripts under the "oxidoreductase" GO-category in the fat body of caterpillars in response to the extended repertoire of plants.

H. melpomene **ortholog to V.** ***cardui*** **transcript**	**Putative function**	**logFC**	**PValue**	**FDR**	**Direction of regulation**
HMEL005163-PA	Pyridine nucleotide-disulphide oxidoreductase	1,10	0,0003	0,0187	Up in response to extended
HMEL007773-PA	Calcium-binding	1,17	0,0001	0,0126	
HMEL008660-PA	Aldehyde dehydrogenase	1,80	0,0002	0,0169	
HMEL015490-PA	Ascorbate-dependent monooxygenase	2,02	0,0006	0,0274	
HMEL010099-PA	RNA helicase, ATP-dependent	2,13	5,39E-05	0,0086	
HMEL015335-PA	Alkyl hydroperoxide reductase subunit C/ Thiol specific antioxidant	2,15	0,0003	0,0184	
HMEL016430-PA	Ribonucleotide reductase	2,15	0,0008	0,0309	
HMEL017529-PA	Aldehyde oxidase/xanthine dehydrogenase	2,40	0,0002	0,0169	
HMEL010191-PA	Glucose-methanol-choline oxidoreductase	2,55	0,0007	0,0294	
HMEL007567-PA	Cytochrome P450, E-class, group I	2,71	0,0002	0,0142	
HMEL006505-PA	Zinc finger C2H2-type/integrase	2,82	0,0008	0,0324	
HMEL017435-PA	Glucose/ribitol dehydrogenase	3,06	1,60E-05	0,0045	
HMEL026007-PA	Cytochrome P450, E-class, group I	3,24	6,25E-05	0,0092	
HMEL026004-PA	Cytochrome P450	3,67	9,31E-07	0,0008	
HMEL010572-PA	Short-chain dehydrogenase/reductase SDR	3,68	7,70E-06	0,0027	
HMEL007720-PA	Multicopper oxidase	3,88	5,61E-06	0,0023	
HMEL009822-PA	Aromatic amino acid hydroxylase	4,27	0,0008	0,0323	
HMEL007718-PA	Multicopper oxidase	8,23	0,0005	0,0258	

HMEL002921-PA	Fatty acid desaturase	-4,20	4,62E-05	0,0080	Down in response to extended
HMEL011526-PA	Fatty acyl-CoA reductase	-3,35	0,0008	0,0323	
HMEL009864-PA	Fatty acyl-CoA reductase	-2,69	0,0002	0,0173	
HMEL008178-PA	NAD(P)-binding domain	-1,95	9,21E-05	0,0120	
HMEL008463-PA	Catalase	-1,68	0,0005	0,0258	
HMEL003184-PA	Flavin monooxygenase FMO	-1,68	0,0001	0,0124	
HMEL008820-PA	D-3-phosphoglycerate dehydrogenase	-1,59	0,0002	0,0141	
HMEL005303-PA	Alcohol dehydrogenase	-1,48	0,0013	0,0435	
HMEL008646-PA	Fatty acid hydroxylase	-1,46	6,66E-07	0,0007	
HMEL011673-PA	Transketolase	-1,28	1,68E-05	0,0046	
HMEL011153-PA	Aldehyde dehydrogenase	-1,27	9,82E-05	0,0121	
HMEL014821-PA	Aldo/keto reductase	-1,27	0,0009	0,0335	
HMEL002896-PA	Glutathione peroxidase	-1,24	0,0006	0,0261	
HMEL012503-PA	3-oxo-5-alpha-steroid 4-dehydrogenase	-1,20	0,0002	0,0169	
HMEL015668-PA	Thioredoxin-like	-1,18	0,0003	0,0209	
HMEL013284-PA	Lactate/malate dehydrogenase	-1,13	0,0002	0,0155	
HMEL015141-PA	Alanine dehydrogenase	-1,10	0,0006	0,0271	
HMEL017418-PA	Alcohol dehydrogenase	-1,09	0,0005	0,0258	
HMEL007836-PA	Pyridine nucleotide-disulphide oxidoreductase	-1,03	0,0006	0,0274	
HMEL006415-PA	Dehydrogenase	-0,98	0,0006	0,0279	
HMEL016639-PA	6-phosphogluconate dehydrogenase	-0,93	0,0016	0,0479	
HMEL011338-PA	NADH:ubiquinone oxidoreductase	-0,84	0,0016	0,0485	
