# Supplementary material for: Evolutionary history of host use, rather than plant phylogeny, determines gene expression in a generalist butterfly
Source: BMC Evol Biol. 2016 Mar 8;16:59. doi: 10.1186/s12862-016-0627-y (PMC4782335; doi:10.1186/s12862-016-0627-y)
Supplement: Additional file 4: — Is a table listing tissue-specific transcripts up- regulated in response to the extended repertoire of plants (PDF 370 kb) [file 12862_2016_627_MOESM4_ESM.pdf]

Additional file 4. List of the five most significantly expressed genes between treatment comparisons within the variables plant use and plant phylogeny. Gene expression data were obtained from different tissues from caterpillars reared on six different plants grouped according to either their evolutionary history of association with nymphalids (plant use) or their relatedness (plant phylogeny). The list includes the *H. melpomene* orthologs to *V. cardui* transcript sequences.

Variable	Comparison	Tissue	*H. melpomene* ortholog	Putative function	Fold change	P-value (adjusted)	Direction of regulation
**Plant use**	**Core vs Extended**	**Fat body**	HMEL012774-PA	calcium-binding protein	3,75	0,005	Up in response to extended
			HMEL008258-PA	Pyridoxal phosphate-dependent transferase	3,45	0,002	
			HMEL010572-PA	Glucose/ribitol dehydrogenase	3,44	4,37E-08	
			HMEL016777-PA	Fibronectin III	3,20	0,004	
			HMEL017157-PA	Fibronectin III	3,17	0,0005	
		**Gut**	HMEL022670-PA	UDP-glucuronosyl/UDP-glucosyltransferase	4,66	1,62E-21	
			HMEL006731-PA	Unknown	4,39	0,001	
			HMEL004447-PA	Cytochrome P450	3,63	7,33E-05	
			HMEL008728-PA	hormone/neurotrans mitter receptor	3,25	0,001	
			HMEL005448-PA	chaperonin (protein folding/ intercellular signalling	2,83	4,53E-07	
		**Malpighian tubules**	HMEL011566-PA	Unknown	4,48	0,0045	
			HMEL007718-PA	Multicopper oxidase	3,85	0,0002	
			HMEL011034-PA	Unknown (containing ferlin domain)	3,56	0,0080	
			HMEL007028-PA	Peptidase	3,22	0,0009	
			HMEL011939-PA	Substrate transporter	2,45	4,12E-05	
		**across all tissues**	HMEL015250-PA	DNA replication factor	6,14	0,0078	
			HMEL005122-PA	Glycogen/starch/alpha-glucan phosphorylase	6,09	0,0026	
			HMEL014753-PA	AAA+ ATPase	3,46	0,0051	
			HMEL003762-PA	Sodium:dicarboxylate symporter	3,22	0,0052	
			HMEL002255-PA	Gustatory receptor	3,17	0,0034	

	**Core vs Extended**	**Fat body**	HMEL012931-PA	unknown	-2,69	0,004	Down in response to extended
			HMEL016187-PA	unknown	-2,82	0,0003	
			HMEL013199-PA	Phosphoenolpyruvate carboxykinase, GTP-utilising	-2,84	6,72E-10	
			HMEL010109-PA	substrate transporter	-2,92	0,002	
			HMEL002921-PA	fatty acid desaturase	-3,81	0,002	
		**Gut**	HMEL011985-PA	sequence-specific DNA binding	-3,03	4,33E-07	
			HMEL015842-PA	heat shock protein	-3,10	0,0007	
			HMEL012915-PA	substrate transporter	-3,34	0,007	
			HMEL025041-PA	Sodium/potassium/calcium exchanger	-3,81	2,15E-06	
			HMEL005076-PA	Cyclin-dependent kinase inhibitor	-3,89	1,92E-07	
		**Malpighian tubules**	HMEL007202-PA	Small GTPase superfamily, Ras type	-2,99	0,0012	
			HMEL006326-PA	Lipocalin/cytosolic fatty-acid binding	-3,01	0,0010	
			HMEL008128-PA	Phospholipase	-3,11	1,30E-05	
			HMEL009687-PA	Peptidase	-3,31	4,64E-06	
			HMEL012910-PA	Substrate transporter	-3,55	1,55E-06	
		**across all tissues**	HMEL013743-PA	Glucose/ribitol dehydrogenase	-1,60	2,17E-05	
			HMEL005076-PA	Cyclin-dependent kinase inhibitor	-1,69	3,81E-05	
			HMEL015840-PA	Alpha crystallin/Heat shock protein	-1,70	1,32E-05	
			HMEL013199-PA	Phosphoenolpyruvate carboxykinase, GTP-utilising	-2,04	4,08E-17	
			HMEL015592-PA	WD40-repeat-containing domain	-5,64	0,0070	
