# Supplementary material for: Evolutionary history of host use, rather than plant phylogeny, determines gene expression in a generalist butterfly
Source: BMC Evol Biol. 2016 Mar 8;16:59. doi: 10.1186/s12862-016-0627-y (PMC4782335; doi:10.1186/s12862-016-0627-y)
Supplement: Additional file 5: — Provides the details of the methodology followed when conducting the oviposition preference and larval performance experiments (PDF 259 kb) [file 12862_2016_627_MOESM5_ESM.pdf]

Additional file 5.

Oviposition preference. Fifteen pairs of butterflies were isolated during copulation and placed in cages with a plastic cup containing a sponge soaked in sucrose solution (mean temp. 26°C; mean RH 49%; LD 18:6). The next day females were placed in a second cage and exposed daily to leaf-cuttings on glass bottles with water from two plants three times per day for five consecutive days. Each paired-choice experiment lasted two hours with breaks of one hour in between in such a way that a given female encountered three different plant combinations during the day. No butterfly had the same arrangement of paired choices during the 5 days. At the end of the third session, plants were removed and males were placed in the cage until the next day. Eggs on plants were counted after each oviposition session and recorded. The total number of choices was fifteen. The preferred plant by butterflies for oviposition was assessed applying the Friedman test, a non-parametric test based on ranks. Two kinds of rank-ordering of plants per female butterfly were performed; one based on proportions (calculated by dividing the number of eggs in a plant by total number of eggs laid by a given female) and the other one based on cumulative frequency of preferred plant for each paired choice where the preferred plant has highest number of eggs. The paired Wilcoxon test with correction for multiplicity was applied as a post-hoc test for multiple comparisons based on Friedman rank sums (Wilcoxon-Nemenyi-McDonald-Thompson) (1).

Larval performance. Two life history traits from caterpillars reared on the different plants were recorded: developmental time to adult emergence and pupal weight. Pupal weight in milligrams was recorded two days after pupation. Growth rate values were calculated by dividing the pupal weight by the number of days to pupation (log-transformed). Normality of the data and homogeneity of variances were assessed by Shapiro-Wilk and Bartlett and Fligner-Killeen tests correspondingly. If needed, the most suitable transformation of the data was derived using the 'box-cox' function for linear models in R 3.0.3. After verifying that the transformed data fulfilled normality and homogeneity of variances, a parametric mixed linear model was performed testing the effect of plant on larval performance. If the treatment effect was significant, a post-hoc Tukey test (p-value cut off= 0.05) for multiple comparisons was performed using R 3.0.3. If normality and homogeneity of variances could not be achieved, data were analyzed using Kruskal-Wallis Chi-squared test and multiple comparisons after non-parametric Kruskal-Wallis test using the package pgirmess in R 3.0.3.

1. Hollander M & Wolfe DA (1999) Nonparametric statistical methods (Wiley, New York ; Chichester) 2. Ed.
