# Supplementary material for: Evolutionary history of host use, rather than plant phylogeny, determines gene expression in a generalist butterfly
Source: BMC Evol Biol. 2016 Mar 8;16:59. doi: 10.1186/s12862-016-0627-y (PMC4782335; doi:10.1186/s12862-016-0627-y)
Supplement: Additional file 6: — Provides the methodology for sequencing of RNA and gene annotation and enrichment of the transcriptional dataset. (PDF 294 kb) [file 12862_2016_627_MOESM6_ESM.pdf]

Additional file 6.

Sequencing of RNA. Survival on the plants determined the extent of family representation in the experiment; there were at least three replicates per feeding treatment. Each replicate consisted of tissue-specific RNA from two caterpillars (combined). The tissues were dissected from cold-anesthetized larvae under phosphate saline solution (pH 7.4, 10 mM), placed in temperature-resistant tubes and snap-frozen in liquid nitrogen. Long-term storage of the samples occurred at -76°C. The tissues dissected from each caterpillar were gut, fat body, Malpighian tubules and labial glands. Therefore, we obtained a total of seventy two (RNA) samples (six plants X four tissues X three replicates). RNA isolation, purification and quantification were conducted following the protocol described previously (1). The quality of RNA was determined using the "Experion" equipment following manufacturer's protocols (Bio-Rad, Hercules, CA). Science for Life Laboratory (SciLifeLab, Sweden) conducted the sequencing of RNA samples. The cDNA libraries (Illumina TruSeq RNA) were sequenced using the Illumina HiSeq 2000 platform using 100-bp paired-end sequencing. We obtained more than 9 million read-pairs from seventy one cDNA libraries sequenced and the transcriptome assembly (TA) of these sequences resulted in 213, 237 transcripts (162,189 components) with a contig N50 of 2,193 bp. Thus, we covered approximately 300x the transcriptome of caterpillars of the species *V. cardui*. We consider of better quality those newly generated transcriptomes with the highest proportion of fully assembled genes in comparison to the nearest known transcriptome. Consequently, we assessed the degree of orthology between the PGS of H. melpomene (clustered, 12,591 genes) and the *V. cardui* TA (clustered, 189,485 contigs). Using the blast-inferred orthologies, we found that about half of the predicted genes for H. melpomene were covered in more than 80% of their sequence by the longest contig (ortholog) from the *V. cardui* TA (Fig. S1A). If the sum of all contigs from the *V. cardui* TA are taken in consideration, more than 70% of those predicted genes for *H. melpomene* are covered in more than 80% of their sequence (Fig. S1B). In other words, 6502 of the *H. melpomene* genes had a single V. cardui TA-contig that covers > 90% of their expected length and 8379 of the H. melpomene genes have > 90% of their expected length covered by multiple *V. cardui* TA-contigs. These findings suggested that the transcriptome generated was of good quality (1) and therefore we continue with the TA for downstream analyses (see figure below).

Gene annotation and enrichment. The annotation of the non-redundant PGS for *H. melpomene* was determined using Blast2GO. The blast2GO annotation was carried out in three steps. First the non-redundant PGS was blasted, using NCBI blast (v2.2.28+), against the nr database, which was used by blast2GO (v2.7.1) (2) to annotate the genes. Second the non-redundant PGS was scanned for known protein motifs, using InterProScan (v5.3-46) (3, 4) with the default database set, which was used by blast2GO to annotate the genes. Finally blast2GO merges the two annotations into one. Using the ontology terms for each identified ortholog, we assessed whether any particular gene category was enriched using topGO (5) using the fisher test. As a background set, we used all Blast-inferred orthologs and the subset examined for enriched GO-terms was the differentially expressed Blast-inferred orthologs. Blast-inferred orthologs were considered to be differentially expressed when, using the read count per Blast-inferred orthologs as input, edgeR (6) in R Bioconductor, using default parameters, assigned them as differentially expressed with an FDR less than 0.05.

A

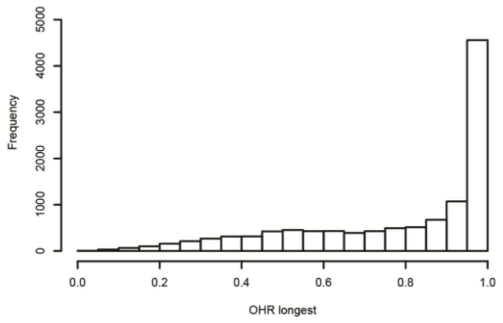

B

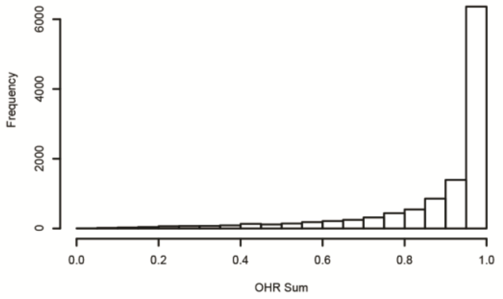

OHR histograms statistics for the *V. cardui* caterpillar transcriptome. The histograms represent the most informative ratios for assessment of transcriptome assemblies: A. the longest TA contig per ortholog; B. the sum of the ortholog length covered by all the TA contigs, which is then divided by the full length of the ortholog.

1. Celorio-Mancera MP, et al. (2013) Mechanisms of macroevolution: polyphagous plasticity in butterfly larvae revealed by RNA-Seq. Molecular Ecology 22(19):4884-4895.

2. Conesa A, et al. (2005) Blast2GO: a universal tool for annotation, visualization and analysis in functional genomics research. Bioinformatics 21(18):3674-3676.

3. Zdobnov EM & Apweiler R (2001) InterProScan - an integration platform for the signature-recognition methods in InterPro. Bioinformatics 17(9):847-848.

4. Jones P, et al. (2014) InterProScan 5: genome-scale protein function classification. Bioinformatics 30(9):1236-1240.

5. Alexa A & J R (2010) topGO: Enrichment analysis for Gene Ontology. R package (2.16.0).

6. Robinson MD, McCarthy DJ, & Smyth GK (2010) edgeR: a Bioconductor package for differential expression analysis of digital gene expression data. Bioinformatics 26(1):139-140.
